# Primary entry trocar design and entry-related complications at laparoscopy in obese patients: meta-analysis

**DOI:** 10.1093/bjsopen/zrad047

**Published:** 2023-06-23

**Authors:** Chimwemwe Miti, Paula Busuulwa, Richard Scott, Hermes Bloomfield-Gadelha

**Affiliations:** Department of Electrical and Electronic Engineering, EPSRC Centre for Doctoral Training in Digital Health and Care, University of Bristol, Bristol, UK; Department of Academic Obstetrics & Gynaecology, Liverpool Women’s Hospital, Liverpool, UK; Department of Engineering Mathematics and Bristol Robotics Laboratory, University of Bristol, Bristol, UK; Department of Medical Physics, University Hospitals Bristol and Weston NHS FT, Bristol, UK

## Abstract

**Background:**

Safe primary entry at laparoscopy could present challenges in obese patients. Various techniques have been proposed in previous studies, however, the characteristics of the actual device utilized may be more influential than the technique in achieving successful abdominal entry in patients with increased BMI.

**Methods:**

This systematic review and meta-analysis included both randomized and non-randomized studies gathered with no date filters from MEDLINE, Embase, Scopus, Web of Science and Clinicaltrials.gov. PRISMA guidelines underpinned the conduct and reporting of the review. The meta-analysis of proportions was conducted using a generalized linear mixed model and analyses included random-effects models. The primary outcome was the proportion of first access vascular and visceral injuries incurred in the process of laparoscopic abdominal surgery in patients with a BMI >30 kg/m^2^. Subgroup analysis was performed for optical *versus* non-optically enabled devices.

**Results:**

In total, 5403 patients were analysed across 13 observational studies with a mean BMI of 45.93 kg/m^2^. In 216 patients from two randomized studies, the mean BMI was 39.92 kg/m^2^. The overall incidence using a random-effects model was 8.1 per 1000 events of visceral and vascular injuries (95 per cent c.i. 0.003 to 0.024). Heterogeneity was statistically significant at *I*^2^ = 80.5 per cent (69.6 per cent; 87.5 per cent, *P<* 0.0001). In a subgroup analysis, a tendency towards reduced injuries when optical devices were employed was observed with one per 100 injuries in these trocars (95 per cent c.i. 0.001 to 0.018) *versus* four per 100 (95 per cent c.i. −0.019 to –0.102) in non-optically enabled devices.

**Conclusion:**

Injuries during primary laparoscopic entry undertaken in obese patient groups are uncommon. Due to considerable heterogeneity in the small number of examined studies, evidence was insufficient and largely of low quality to ascribe differences in the incidence of injuries to the characteristics of the primary entry trocar utilized.

## Teaser

This systematic review and meta-analysis reports the frequency of resultant visceral and vascular complications, at first entry and by entry device type, in specified obese classes of patients undergoing laparoscopic abdominal surgery.

## Introduction

It is important that the development of devices assisting safer laparoscopic primary entry is accelerated^1^ to address the difficulties in patients with challenging characteristics. As a minimally invasive technique, laparoscopy is regarded as an essential component of Enhanced Recovery After Surgery (ERAS) programmes, especially in gynaecology and general surgery, and its use, if possible, is strongly recommended^2^. Nonetheless, laparoscopy carries specific complications^3,4^ and establishment of primary access can be hindered by entry challenges, which may cause considerable morbidity in patients^5–7^. The initial access into the abdominal cavity first requires the introduction of an entry device usually in the form of a trocar or a Veress needle to allow the creation of a pneumoperitoneum and intra-abdominal working space. Entering the abdominal cavity is considered risky largely due to its blind nature and dependence on the surgeon’s ability to ‘sense’ or visually identify peritoneal entry and it has led to several studies discussing the safety constraints^8^. Previous reviews and trials exploring laparoscopic abdominal entry complications have dealt largely with the entry technique rather than the entry device^9,10^. However, these studies have excluded important groups of patients, such as obese individuals or those with previous abdominopelvic surgery^11,12^.

The extent of various complications in published literature during first entry is often underestimated, as highlighted by a review of medicolegal claims in the Netherlands involving laparoscopic entry^13^, which as a result, could not establish precise estimates. On the other hand, a Finnish study where a policy of ‘no medical fault’ compensation scheme exists, may have come close to documenting the incidence of resultant laparoscopic entry injuries^14^. Evidence supporting the reduction of primary access complications in such patients therefore remains inadequate. The challenge in investigating the contribution of a primary access device to resultant complications is due to the variety of entry devices and techniques utilized by surgeons of differing specialism, experience and preferences. These reasons further underlie difficulties encountered in surgical trial design and execution^15^.

Traditionally, entry devices have included the Veress needle, first invented by Janos Veress in 1932 for the purpose of treating pulmonary tuberculosis and later adapted by Palmer for gynaecological laparoscopy^16^ to create the pneumoperitoneum^17^. Unfortunately, Veress needle insertion (VNI) and the subsequent creation of a pneumoperitoneum is not without surgical risk^18^. Beyond the Veress needle, Hasson or direct entry trocars each bearing unique identity to the manufacturer and whether bladed, blunt or shielded, have comprised another major group of entry devices whose use in laparoscopic procedures has evolved over time. Other novel devices such as the EndoTIP (Endoscopic Threaded Imaging Port, Karl Storz Endoscopy, Tuttlingen, Germany)^19,20^ and Kii Fios (Applied Medical, Rancho Santa, Margarita, CA, USA) optical trocar systems^21–24^ have also been developed to solve some of the commonly encountered obstacles in establishing primary access. The latter device simultaneously permits carbon dioxide insufflation during entry. Optical access devices (bladed or non-bladed) allow direct visualization of the anatomical layers as the abdominal wall is being traversed and offer more than just surgical tactile sensation. These include Versaport (Medtronics, Minneapolis, MN, USA)^22^, Optiview (Ethicon, Cincinnati, USA)^25–27^, EndoPath/EndopathXcel (Ethicon, Cincinnati, OH, USA)^28,29^, Visiport Plus (Tyco United States Surgical, Norwalk, CT, USA)^30–32^ and Versaport Plus (Covidien, Dublin, Ireland)^33^. Other devices evaluated in experimental settings aside of live surgery include an ultrasonically activated trocar system^34^ and a dilating missile trocar which has not yet reached market^35^.

The aim of this systematic review and meta-analysis was therefore to determine the frequency of entry-associated injuries in obese patients undergoing laparoscopic surgery and assess whether this is related to the characteristics of the primary entry trocar used. It hypothesizes that the design of the trocar or the characteristics of its tip, rather than individual entry techniques^36–38^, influence complications at laparoscopic entry.

## Methods

### Registration and protocol

Trial Registration of the study protocol and study registration were not achieved prior to commencing the systematic review.

### Study design and selection process

The study was designed as a systematic review and meta-analysis of published observational and RCTs to extract collective evidence on the incidence of laparoscopic primary entry injuries in obese patients according to the design of entry device used. A summary of the study according to the PICO model (Population, Intervention, Comparison, Outcomes)^39^ is shown in *Table S1*.

The conduct of the review followed reporting guidelines of observational studies (MOOSE, Meta-analyses of Observational Studies)^40^ as well as that for systematic reviews and meta-analyses (PRISMA)^41^. Risk-of-bias plots were constructed using published guidance^42,43^ and calculations respectful of the different study types proceeded using appropriate meta-analytic packages.

Retrospective or prospective studies with no date filters and concerning human laparoscopy were included if published in English and providing participant obesity class. Case reports, conference articles or isolated abstracts were excluded.

Two authors independently undertook a comprehensive literature search including PubMed, MEDLINE, Embase, Scopus, Cochrane Library, Web of Science and Clinicaltrials.gov for relevant articles.

A search strategy used each of the following term combinations in each database and register:

laparoscopy AND entry AND obesity AND trocar AND complications; laparoscopy AND entry AND complications AND surgeon; laparoscopy AND entry device AND complications; laparoscopy AND entry AND complications AND surgical training; entry trocar AND complications; trocar AND tip AND design AND laparoscopy; trocar AND laparoscopy AND novice.

The search strategy followed PRISMA guidelines. Following the initial search according to the listed terms, screening of study titles and abstracts proceeded ensuring selection of studies where the measured BMI was documented alongside the type of entry trocar assessed. Eligible articles were screened by the manual review of full texts.

### Study risk-of-bias assessment

The ‘ROBINS-I’ tool (Risk of Bias In Non-randomised Studies of Interventions) was implemented in screening the case series^44^, while the ‘Risk of Bias 2 (Rob-2)’ tool was employed to assess the bias risk of the RCTs^45^.

### Effect measures

The choice of effect measures was straightforward in the RCTs and the dichotomous results between the intervention groups were compared using Peto Odds Ratio^46^. When combined with the observational group of studies, the effect size of all the outcomes was an expression of the proportion of events (injuries) in the sample. Individual study weights as well as the weighted effect sizes were all computed.

### Outcomes of interest

The primary outcome was incidence of all vascular or visceral injuries in obese patients undergoing laparoscopic surgery. Of note, this systematic review adopted an all-encompassing term of ‘visceral or vessel injuries’ regardless of the severity, as the predefined outcome.

Two subgroups analysed posthoc were use of optical or non-optical trocars and trocar entry with or without the establishment of a prior Veress needle pneumoperitoneum.

### Data collection and statistics

Data points included the study author and publication year, laparoscopic procedure(s), patient sample size, mean BMI, duration of study and follow-up if applicable, specific device used with the manufacturer details (bladed/unbladed, optical/non-optical), method of establishing the pneumoperitoneum, number of surgeons involved, mean entry time where measured, site of abdominal entry, size of incision and crucially, explicit nature and number of injuries.

Data items collected from each study article were manually entered into a data sheet in Microsoft^®^ Excel (Version 2109)^47^. Derived data was stored as Comma Delimited (*.csv) files ready for importation into ‘R’ software for statistical analysis.

All statistical computations were performed in ‘R’ software for statistical analysis (R version 4.1.1, 10/08/2021, ‘Kick Things’)^48^ with various ‘R’ meta-analytic packages utilized in constructing the forest plots and presenting statistical results^49^. The meta-analytic method for proportions was applied to the analysis of the outcome of interest in both the randomized and non-randomized studies, while a Peto Odds Ratio was used specifically to calculate the odds of the rare outcome (vascular or visceral injury) according to device utilized across the RCTs. The generalized linear mixed model (GLMM) was used in this meta-analysis of proportions. A random-effects model was implemented in the production of forest plots due to study inhomogeneity. Heterogeneity was calculated using the DerSimonian–Laird procedure^50^ in the ‘-meta’ package (a random intercept logistic regression model) alongside a maximum likelihood estimator for *Tau*^2^. For the random-effects model, Hartung–Knapp adjustment was made through Logit transformations of the raw number of events (injuries). Confidence intervals for the individual studies were calculated using Clopper–Pearson analysis with a continuity correction of 0.5 in those studies that had zero cell frequencies as some studies had zero events of entry injuries. Confidence intervals were set to 95 per cent. The same meta-analytical method was used in finding study outliers. All graphs were automatically produced using the appropriate packages in ‘R’ Studio, or where needed, codes were obtained from freely available online resources^51–53^.

In all results reported 95% c.i. was used and a *P* value of *<*0.01 signified statistical significance. Heterogeneity (*I*^2^) of 50 per cent was considered moderate and substantial when computed at or above 75 per cent. The ROBINS-1 tool was the method used to assess the level of certainty of the evidence provided in the observational studies, whereas the ‘GRADE’ system (Grading of Recommendations, Assessment, Development and Evaluations) was applied to the certainty rating of the RCTs with a result of ‘moderate’ in the latter.

## Results

### Study selection and characteristics

**Fig. 1:**
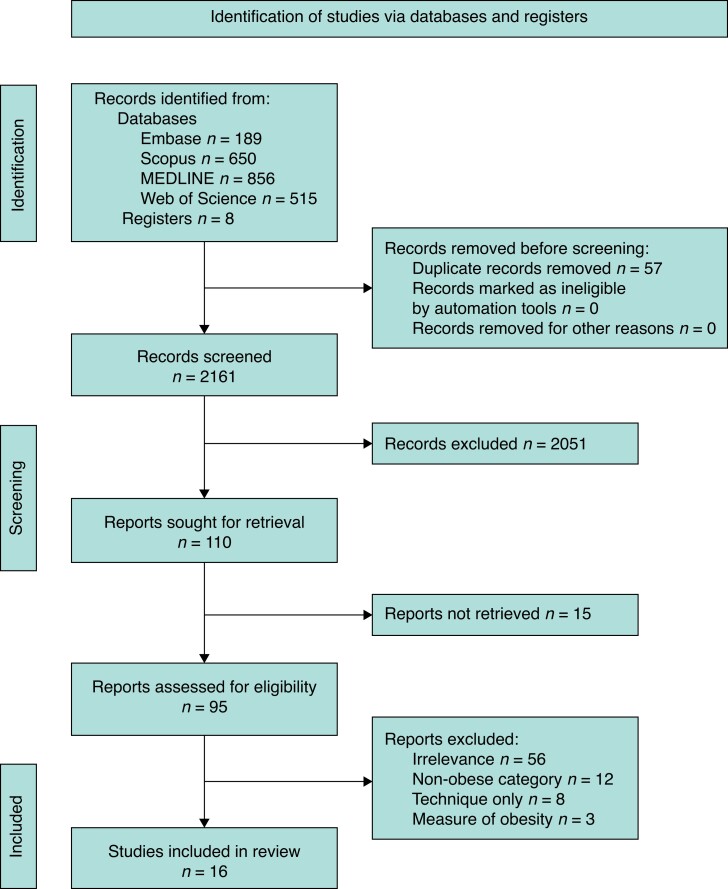
PRISMA flow diagram Records excluded: on abstract review entry criteria unmet. Reports not retrieved: full texts were unavailable

*Figure 1* reports the PRISMA flow diagram with the selection process. Overall, 13 observational studies^20–24,29,30,32,54–58^ and three RCTs^33,59,60^ were included in the systematic review but exclusively a total of 15 in the final statistical analysis^20–24,29,30,32,33,54–59^. One RCT^60^ had to be excluded from the meta-analytic assessment as it compared devices dissimilar to those employed in the other RCTs. Additionally, the number of surgeons involved, participant age range and gender were not consistently provided across all studies and hence not reported in the final analysis.

### Risk of bias in studies

**Fig. 2:**
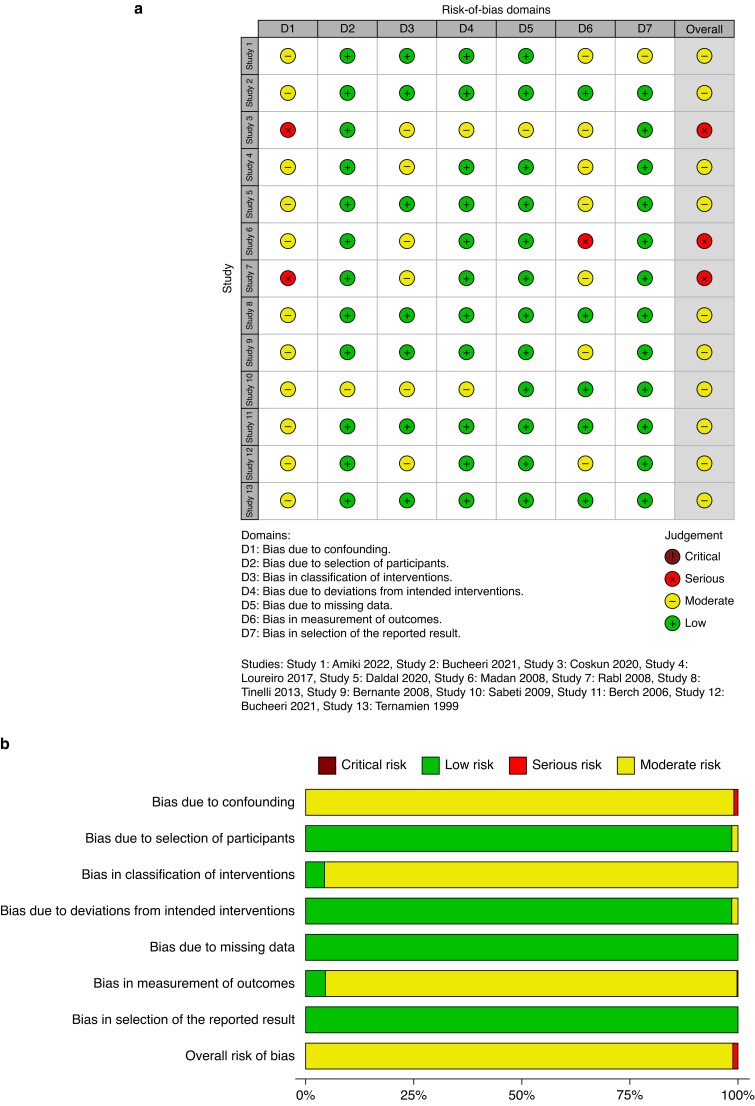
**a** Traffic light plot: bias risk in individual observational studies. **b** Bias risk: observational studies of laparoscopic surgery in obese patient groups

**Fig. 3:**
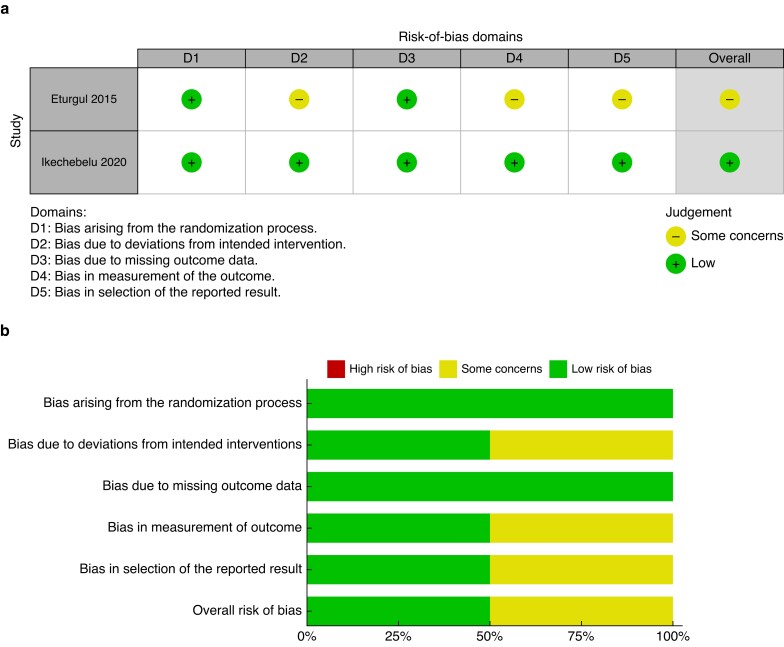
**a** Traffic light plot of the risk of bias in each randomized study domain. **b** Collated weighted bar plot of the risk of bias in the randomized studies

When analysing the manuscripts for the risk of bias, all case series had potential for confounding factors. Moreover, with differences in the number of operating surgeons alongside variability in the access site of primary entry and method of establishing the pneumoperitoneum, bias risk was moderate in the observational studies (*Fig. 2*). *Figure S1* details the assessment outcome of the raw data before producing the plot. A traffic light plot of the assessed bias in the RCTs is illustrated in *Fig. 3*.

**Fig. 4:**
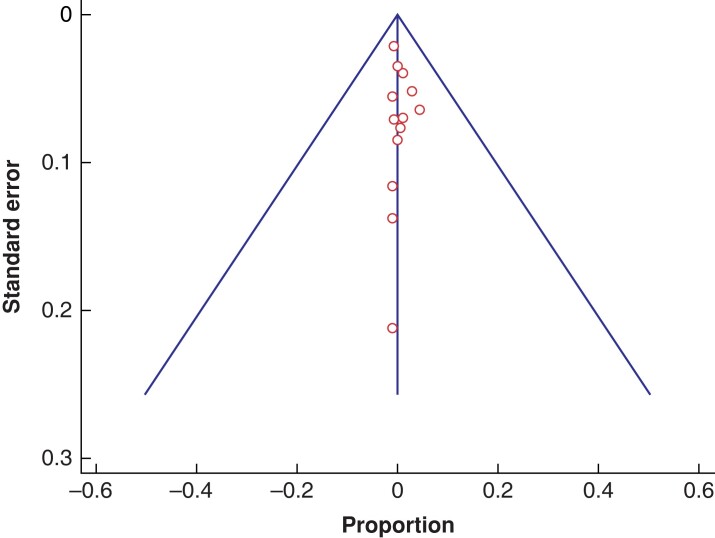
Funnel plot showing the absence of publication bias

It should be noted that some studies had zero proportions of events reported with the device used to achieve primary entry in terms of any resultant visceral or vessel injury but ‘minor’ complications could still have occurred and not been published due to subjectivity in assigning study outcomes as reportable injuries. Nonetheless, the funnel plot in *Fig. 4* demonstrates symmetry and publication bias may not be present, although a confirmatory Egger’s test^61^ was not performed for this specific purpose.

In the plot displayed, the size of trials is plotted against the individually reported effect size (by proportion). It shows a scattering of studies remarkably close to and on either side of the overall effect line. The open circles illustrate the statistical non-significant results of the studies represented.

With respect of the certainty of evidence, for both the RCTs and non-randomized studies, a grading of ‘moderate’ was assigned (*Fig. S2*).

### Systematic review

One RCT^60^ met the selection criteria but was excluded from the meta-analytic calculations as the devices compared in this study were dissimilar to those assessed in the other two RCTs^33,59^ and so comparisons across all studies would not be possible.

**Fig. 5:**
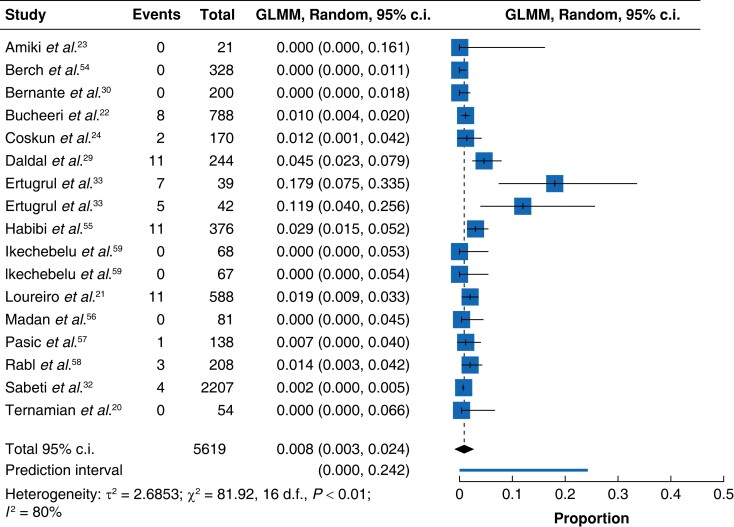
Incidence by proportion of primary entry-associated injuries in randomized and observational studies involving obese patients GLMM, Generalised Linear Mixed Model.

A total of 5619 patients were entered into the final meta-analysis with a resulting 63 visceral or vessel injuries (51 resultant injuries from the observational studies and 12 across the RCTs) in patients with a BMI > 30 kg*/*m^2^ on initial abdominal entry during abdominopelvic laparoscopic procedures (*Fig. 5*). A detailed inspection of the analysed studies reports that a total number of 5403 patients made up the observational study group of 13 studies with a mean BMI of 45.93 kg*/*m^2^.

Of note, 54 patients in a single surgical case series of 234 patients incorporated BMI ranges from *<*30 to *≥*35 kg*/*m^2^ so a mean BMI could not be readily calculated and therefore averaged as 35 kg*/*m^2^ for convenience. A total of 216 patients with a mean BMI of 39.92 kg/m^2^ made up the randomized group of two studies.

For all studies^20–24,29,30,32,33,54–59^, the pooled incidence by proportions of visceral or vessel injury in the selected studies using a random-effects model is 8.1 per 1000 events of visceral and vascular injuries (95 per cent c.i. 3–24 per 1000 cases). With the *I*^2^ statistic computed at 80 per cent, heterogeneity amongst studies was substantial. Effect sizes varied amongst the studies and this was statistically significant (*P<* 0.01). The associated forest plot of all the surgical trials is shown in *Fig. 5* and reports the incidence by proportion of visceral or vascular injury incurred during the course of laparoscopic bariatric surgery or operative laparoscopic procedures in obese patient groups using augmented trocars with or without prior abdominal insufflation from a Veress needle. Only one study solely used a Veress needle^57^. Studies with squares to the left of the line of null effect^20,23,30,32,54,56,59^ show a low incidence of the outcome of interest (vascular or visceral injuries) while those to the right^21,29,33,55,58^ reveal a greater incidence. Confidence intervals of individual studies are wide and most studies are around the line of null effect. Finally, it can be observed that the horizontal tips of the diamond line cross the line of no effect, suggesting the combined findings are unlikely to be statistically significant.

One RCT^33^ compared the outcome of entry injury using a bladed non-optical entry trocar (Versaport Plus, Covidien, Auto Suture, Covidien, Mansfield, MA, USA) in 39 patients with an average BMI of 45.8 kg*/*m^2^ to a Veress needle group of 42 patients with a mean BMI of 45.2 kg*/*m^2^. The second RCT^59^ compared this outcome between a group of 68 patients with an average BMI of 34.8 kg*/*m^2^ in which a bladed non-optical trocar (OM Surgicals, Mumbai, India) was used to a Veress needle group of 67 patients with a mean BMI of 33.9 kg*/*m^2^. The third RCT^60^ compared entry injuries between a group of 108 patients with a mean BMI of 34.9 kg*/*m^2^ in which a bladed optical trocar was used (EndoPath or EndoPath Xcel; Ethicon Endosurgery, Cincinatti, OH, USA) to a group of 116 patients of average BMI 35.1 kg*/*m^2^ in which an open Hasson entry trocar was used (Ethicon Endosurgery). Because the Veress needle was not used as a comparator as seen in the other two RCTs^33,59^, this study was left out of the meta-analysis.

**Fig. 6:**
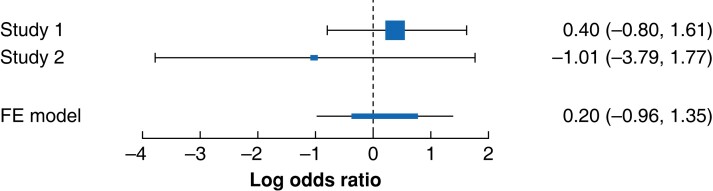
Forest plot of the randomized studies using Peto Odds Ratio RCT results using Peto Odds Ratio. Study 1 = Ertugrul 2015, Study 2 = Ikechebelu 2020. FE, fixed effects.

Separately analysing the two RCTs^33,59^ where entry-related injuries in the obese groups were compared between the Veress needle (VN) and Versaport Plus or OM-Surgical Direct entry trocar, the overall odds of major vascular and visceral injuries using the Peto method was *<* 1 (Peto OR 0.20, 95 per cent c.i. −0.96 to 1.35; two RCTs; *n* = 216). There was little heterogeneity in the RCT group but with only two studies entered into the analysis, cautious interpretation is advised. The small square in study 2^59^ of *Fig. 6* depicts the small sample size involved in this study. It can be further observed in *Fig. 6* that there is no statistically significant difference between visceral and vascular injuries in the groups using the direct trocars (Versaport Plus or ‘OM’ Surgical) and the Veress needle in this particular analysis. For all studies ^20–24,29,30,32,33,54–59^, when combined, entry device did not show an overall impact on complications and so inferences that the design of trocar tips may not be associated with a lower incidence of injuries cannot be stated. Another RCT^53^ reported seven injuries out of 116 patients whose primary entry was accomplished using Ethicon’s Open Hasson Trocar. There were no injuries in the EndoPath or EndoPath Xcel group.

*Table 1* displays studies employing optical first entry devices and *Table 2* displays studies employing non-optical first entry devices, whilst *Table 3* outlines the study characteristics. The nature of these injuries and the specific procedures in which they occur are presented in these tables by study author and show the mean BMI of participants.

**Table 1:** Studies employing optical entry devices and related injuries

Entry trocar (manufacturer)	Author and year	Procedure	Mean(s.d.) BMI (kg/m^2^)	Injury (*n*)	Injury type
Kii Fios Optical (Applied Medical)	Amiki 2022^23^	LSG	41.9(6.5)	0	None
	Bucheeri 2021^22^	SG, GBP, RYGB, BPD	45.9*	4	Mesenteric vessel, liver laceration
	Coskun 2020^24^	SG, RYGB	45.8(6.0)	2	Omental laceration
	Loureiro 2017^21^	SG, GB, RYGB	42.4*	11	Liver laceration, omental tear
EndopathXcel (Ethicon)	Daldal 2020^29^	SG, GBP	46.2*	11	Omental injury, small bowel mesentery
	Madan 2008^56^	Bariatric unspecified	47.0(6)	0	None
	Rabl 2008^58^	GBP, AGB, SG, CC, NFP	45.6*	3	Omental vessel, small bowel mesentery
	Tinelli 2013^60^	Bariatric unspecified	34.9(5.1 9) (DOE group) 35.1(4.9) (OHT group)	0	None
VisiportPlus (Tyco US Surgical)	Bernante 2008^30^	LAGB, SG, GBR	48.0*	0	None
Visiport (Covidien)	Sabeti 2009^32^	GBP, LAGB	42.0* (LAGB) 47 *(GBP)	4	Mesenteric vessel
Optiview (Ethicon)	Berch 2006^54^	RYGB	49.7*	0	None
Versaport (Medtronics)	Bucheeri 2021^22^	SG, GBP, RYGB, BPD	45.9*	4	Mesenteric vessel, omental vessel, liver laceration
EndoTIP (KarlStorz)	Ternamien 1999^20^	Diag.lap or Op.lap	35.0*	0	None
Total (%)				39 (61.9)	
Mean(s.d.) BMI kg/m^2^ 43.87(4.57)	Mean(s.d.) injuries 3.0(3.94)				

*s.d. unavailable. BMI data is presented as mean(s.d.) where available. LSG, laparoscopic sleeve gastrectomy; SG, sleeve gastrectomy; GBP, gastric bypass; RYGB, Roux-en-Y gastric bypass; BPD, biliary pancreatic diversion; LAGB, laparoscopic gastric banding; GB, gastric band procedures; CC, cholecystectomy; NFP, Nissen Fundoplication; DOE, direct optical entry; OHT, open Hasson technique; Op.lap, operative laparoscopy; GBR, gastric band removal; Diag.lap, diagnostic laparoscopy.

**Table 2:** Studies employing non-optical primary entry devices and related injuries

Entry trocar (manufacturer)	Author and year	Procedure	Mean(s.d.) BMI (kg/m^2^)	Injury (*n*)	Injury type
Versaport Plus Auto Suture (Covidien)	Ertugrul 2015a^33^	SG, RYGB	45.8(5.9)	7	Omental, abdominal wall bleed, TMC
Versaport (US Surgical)	Pasic 1999^57^	Diag.lap or sterilization	44.0*	1	Inferior mesenteric artery
Veress Needle	Ertugrul 2015b^33^	SG, RYGB	45.2(6.5)	5	Omental, abdominal wall bleed
	Ikechebelu 2020b^59^	Lap Dye Test	33.9(2.0)	0	None
*Disposable shielded trocar	Habibi 2017^55^	LSG	48.7*	11	Liver, subcutaneous emphysema, omental
Total (%)				24(38.1)	
Mean(s.d.) BMI kg/m^2^ 42.12(6.11)	Mean(s.d.) injuries 4.0(4.47)				

*s.d. unavailable. BMI data is presented as mean(s.d.) where available. SG, sleeve gastrectomy; RYGB, Roux-en-Y gastric bypass; TMC, transverse mesocolon; Diag.lap, diagnostic laparoscopy; Lap Dye Test, laparoscopic chromotubation test; LSG, laparoscopic sleeve gastrectomy.

**Table 3:** Entry device design characteristics and related injuries

Primary entry device	Manufacturer	Trocar characteristics tip design	Injuries per device (*n*)	Injuries per device (%)
Kii Fios	Applied Medical	Optical, insufflating, conical, bladeless	17	32.7
EndopathXcel	Ethicon Endosurgery	Optical, conical, bladeless	14	26.9
VisiportPlus	Tyco US Surgical	Optical, blunt obturator, enclosed crescent blade	0	0
Visiport	Covidien	Optical, triggered spring-loaded blade	4	7.7
Optiview	Ethicon Endosure	Optical, conical, bladeless	0	0
Versaport	US Surgical	Non-optical, bladed, dolphin nose	1	1.9
VersaPort	Medtronic	Optical, dolphin nose, bladeless	4	7.7
Versaport Plus	Medtronic	Optical, bladeless, conical	7	13.5
EndoTIP	KarlStorz	Optical, threaded cannula, blunt open distal end	0	0
Veress needle	Various	Non-optical, sharp bevelled tip, blunt stylet	5	9.6
Total Mean(s.d.) injuries 5.2(5.98)			52	100

11 additional injuries occurred from a trocar with unavailable details (Habibi *et al^55^*).

*Table 3* further tabulates the entry devices used across all studies according to manufacturer and the device design. The majority of studies employed primary entry devices designed to allow optical views on insertion, which could be due to the need to reduce the level of risk involved in achieving entry in obese patients.

**Fig. 7:**
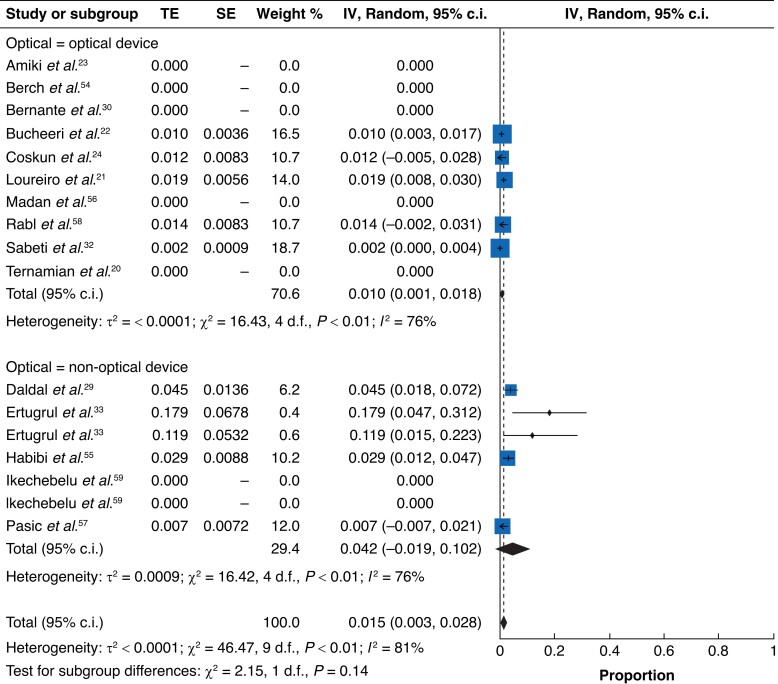
Forest plot of optical entry device subgroup (optical *versus* non-optical entry)

**Fig. 8:**
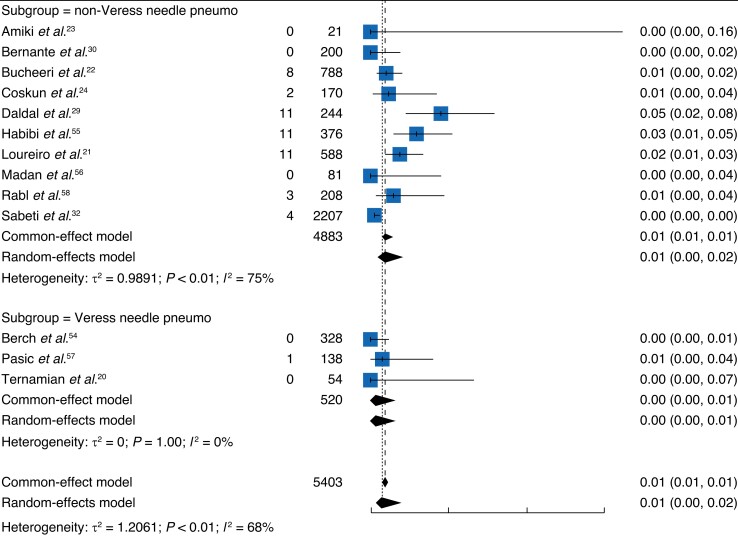
Forest plot of pneumoperitoneum subgroup (establishment of pneumoperitoneum before entry trocar insertion: non-Veress needle pneumo *versus* Veress needle pneumo)

Although subgroup analyses were not defined *a priori*, the results were assessed according to optical-assisted device use (*Fig. 7*)^20,23,30,32,54,56,59^ and pneumoperitoneum introduction before primary entry trocar insertion (*Fig. 8*)^21,29,33,55,58^. This examination appeared to show that first, creation of a pneumoperitoneum with a Veress needle before entry may result in fewer injuries and second, fewer injuries occurred in the optical entry device group compared with the non-optical device group. Caution should be taken with this observation because of the lack of statistical significance and the exceedingly small numbers of studies forming part of the subgroups.

### Results of individual studies

Trocar types utilized in the studies were carefully documented. All the entry devices employed in the two RCTs^33,59^ analysed were bladed and non-optical. With the observational study group of 13 studies^20–24,29,30,32,54–58^, ten studies utilized bladeless trocars^20–24,29,54–57^, while three studies employed bladed trocars^30,32,58^ (these were also optical). Furthermore, concerning visually assisted entry, 11 studies had some form of optical entry trocars^20–24,29,30,32,54,56,58^ while two did not^55,57^ (these were bladeless). Three studies insufflated the abdomen with a Veress needle before primary entry^20,54,57^. The RCTs^33,59^ were additionally analysed separately to the observational studies. Overall, 12 incidents of vascular or visceral injury occurred in the RCTs of 216 participants whilst 51 events occurred in the observed group of 5403 surgical case series. It must be noted that the BMI range in all studies was higher than 30 and mostly in the morbid class (*>*40 kg*/*m^2^), with one exception^20^.

## Discussion

This systematic review and meta-analysis has provided an estimate from the published studies, not previously quantified, of the frequency of entry-related vascular or visceral injuries by trocar design in obese patients undergoing laparoscopic abdominal or pelvic surgery at 8.1 per 1000 patients.

Bariatric procedures made up the majority of operations by virtue of the study design which demanded obese patients. We believe that this is the first study that has collated primary entry-associated injuries in laparoscopic procedures undertaken in obese patients.

However, the weight of evidence presented has not been able to support the design of any particular trocar or the ideal configuration of its tip as a factor in reducing entry complications. While most studies included were observational, these nonetheless provide a glimpse into the nature of complications in high-risk patients who have hitherto been excluded from such studies. Issues of statistical heterogeneity and bias arise with this non-randomized study approach and so interpretation of results is best conducted with this in mind. Pivotal to the daily execution of laparoscopy, this meta-analysis has provided frequencies of laparoscopic first entry complications no worse than those seen in non-obese groups, occurring in 8.1 per 1000 cases.

Outcomes were investigated as a single primary outcome of trocar-associated complications at initial entry. Interestingly, the results from the subgroup analysis, although not statistically significant, call for further investigation. On the other hand, inference of conclusions from the only two RCTs^33,59^ making up the analysis, which themselves did not use the same make and model of the direct entry trocar, may be misleading. In summary, no effect on outcome by trocar type used in laparoscopic obese surgery was observed. The RCTs revealed no significant advantage over the Veress needle of Versaport Plus or ‘OM’ Surgical trocars, in terms of major visceral and vascular injuries. Subgroup analyses of the assessment of injury by trocar tip and trocar characteristics did not provide anticipated answers to the study question, likely due to the small number of studies involved. Three of the case series^32,57,58^ incorporated trainee surgeons (exact grades undefined), whilst the number of surgeons in each study was not always made clear. It is worth noting that a surgeon’s preference for devices may differ.

In particular, four of the observational studies reported a zero rate of injuries^20,30,54,56^. All of these utilized optical trocars, only one study utilized a bladed trocar^30^ and two of these insufflated the abdomen with a Veress needle before entry^20,54^. One of the RCTs^33^ reported more complications, which may be attributed to the higher BMI classes involved in their bariatric procedures and cannot be said of the others^59,60^, as both carried out relatively minor gynaecological laparoscopic procedures in less obese patients. However, whether major or minor surgery, primary entry is the first step and so it is reasonable not to exclude minor procedures as obesity class is what appears to be implicated in laparoscopic entry complications^57^.

Noting incidences of injury from other studies, one author^62^ cited major vessel injury occurring in 0.9 per 1000 cases and visceral injury in 1.8 per 1000 cases with the various trocars assessed. However, this review only had 654 participants in the RCTs groups. Another major review reporting outcomes of techniques^9^ also examined and found no difference in visceral (Peto OR 0.95) or vascular injury (Peto OR 0.14) whether radially expanding or cutting trocars were utilized. Overall, they reported 2 per 1000 and 4 per 1000 of vascular and visceral injury respectively in closed entry techniques, whereas these occurred in 3 per 1000 and 2 per 1000 cases when open entry techniques were considered. The finding here reported in the bariatric patient group of collective visceral and vessel injury of 8.1 per 1000 patients, whilst being modestly higher, is expected due to the high risk this group presents on attempt to establish primary access. Finally, in a large Scandinavian registry of obese patients undergoing laparoscopic Roux-en-Y bypass and where optical trocars were used as the primary entry device^63^, an overall rate of 0.07 per cent cases of intra-abdominal injuries was noted. These results are not dissimilar to what has been presented here, despite being conducted in patient groups that were not strictly in the obese classes.

While trocar characteristics have been the objective of this review, one group of authors described modifications to routine entry in obese individuals that may reduce complications^64^. Similarly, a study introduced an umbilical elevation technique^65^ whereas another outlines alterations to closed umbilical entry where primary insertion of a ‘Step Veress needle’ and pneumoperitoneum creation is followed by removal of the inner Veress and insertion of a bladeless trocar (‘Step trocar’) and expansion of the entry site without removal of the outer shield^66^.

The strengths of this review lie in the provision of incidences in obese groups of laparoscopic primary entry complications. Furthermore, a thorough search for applicable studies and the inclusion of studies with clearly identified devices in well-defined obese classes of participants was undertaken. An effort to incorporate both observational and RCTs was also made. Statistical analyses were performed according to meta-analysis requirements and reported accordingly. Findings were also comparable to previous studies assessing entry-associated injuries during laparoscopic surgery across varied patient groups. The main limitation is the small number of studies available, especially RCTs. The overall quality of evidence was also low. In this regard, the need for high-quality surgical data is obviated.

Future work could focus on engineering devices that are enabled with appropriate technology to further enhance the probability of safe entry void of complications. As an integral entity of the factors assuring surgical safety, these designs should take advantage of novel technologies such that partially automated entry may become a future possibility.

## Supplementary Material

zrad047_Supplementary_Data

## Data Availability

This systematic review has been reposited to the Systematic Review Data Repository (SRDR) under Record ID 3341 (https://srdrplus.ahrq.gov). Additionally, all data alongside the programming codes used to generate the various charts are freely available on request from the corresponding author. Some of this data can also be accessed from the supplementary items folder.
