# Supplementary material for: Primary entry trocar design and entry-related complications at laparoscopy in obese patients: meta-analysis
Source: BJS Open. 2023 Jun 23;7(3):zrad047. doi: 10.1093/bjsopen/zrad047 (PMC10289830; doi:10.1093/bjsopen/zrad047)
Supplement: zrad047_Supplementary_Data [file zrad047_supplementary_data.docx]

**Title**

**Primary Entry Trocar Design and Entry Related Complications at Laparoscopy in Obese Patients: Meta-Analysis**

**Authors**

^1^Miti. C

^2^Busuulwa. P

^3^Scott. R

^3^Gadelha. H

^1^Affiliation University of Bristol, Queen’s Road, BS8 1QU

^2^Affiliation Liverpool Women’s Hospital, Crown Street, L8 7SS

^3^Affiliation University of Bristol, Queen’s Road, BS8 1QU

**Corresponding author.** C. Miti, University of Bristol, Queen’s Road, BS8 1QU **ORCID ID** 0000-0001-8309-269X

**Supplementary Materials - Index**

**Supplementary Tables and Figures**

**Table S1.** *Pag.2*

**Fig. S1** *Pag.2*

**Fig. S2** *Pag.2*

**Supplementary Tables and Figures**

**Table S1.** Study Question Using PICO Model

| P | Obese patients undergoing laparoscopic surgery |
| --- | --- |
| I | Various primary entry devices |
| C | No comparator (observational studies) or as specified (RCTs) |
| O | Any vascular and visceral injuries |

**Fig. S1. Robins-I Bias Assessment Observational Studies**

**
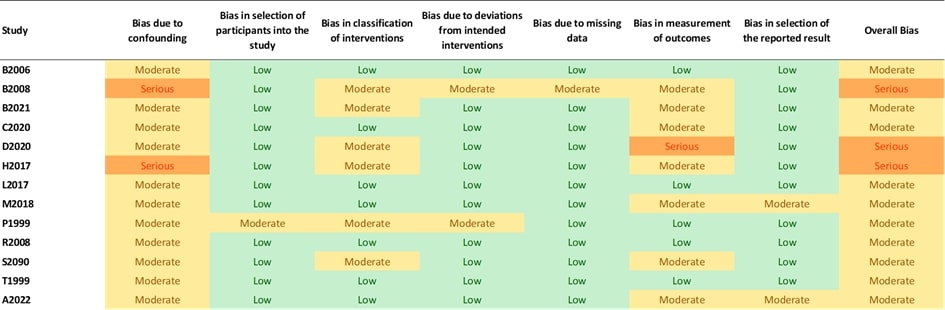
**

**Fig. S2. GRADE assessment of the included RCTs**

**
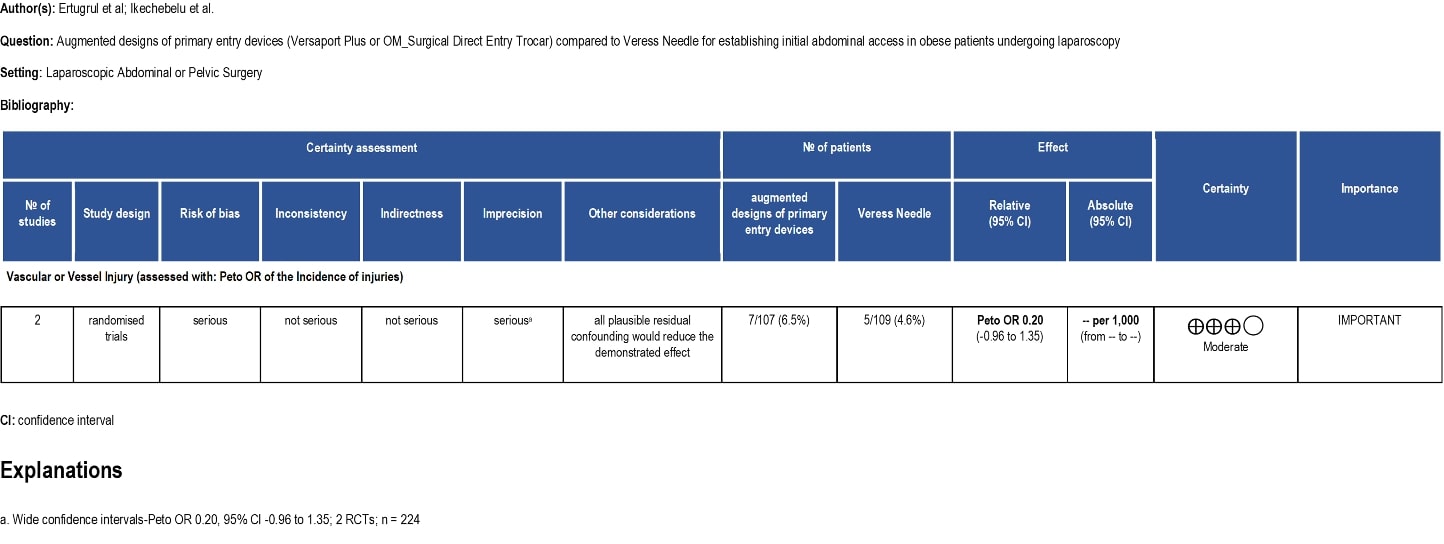
**
